# Acid Rain Phenomenon in Niger Delta Region of Nigeria: Economic, Biodiversity, and Public Health Concern

**DOI:** 10.1100/tsw.2008.47

**Published:** 2008-08-28

**Authors:** J. K. C. Nduka, O. E. Orisakwe, L. O. Ezenweke, T. E. Ezenwa, M. N. Chendo, N. G. Ezeabasili

**Affiliations:** ^1^ Pure and Industrial Chemistry Department, Nnamdi Azikiwe University, Awka, Anambra State, Nigeria; ^2^ Toxicology Unit, Department of Pharmacology, Nnamdi Azikiwe University, College of Health Sciences, Nnewi Campus, Nnewi, Anambra State, Nigeria; ^3^ Chemistry Department, Anambra State University, Uli, Nigeria; ^4^ Department of Environmental Management, Nnamdi Azikiwe University, Awka, Anambra State, Nigeria

**Keywords:** oil exploration, gas flare, acid rain, precursor gases, Niger Delta region, Nigeria

## Abstract

Rain samples were collected from Warri and Port Harcourt, two major oil-producing cities of Nigeria in April-June, July-August, and September-October 2005 and 2006. Awka, a “non-oil” city was used as control. Samples were collected from three points, using clean plastic basins fastened to a table, 2 m above ground level and 115 m away from tall buildings and trees. Water samples were filtered and acidity determined using digital pHmeter. The results show that the rain samples were acidic. The pH values for the 2 years under study show that the rainfall in Warri was more acidic than that of Port Harcourt. Oil exploration and other anthropogenic sources may be responsible for the acid rain in the Niger Delta region of Nigeria.

## INTRODUCTION

The Niger Delta region of Nigeria is the most naturally endowed part of the country, from housing the oil and gas reserves that drive the nation's economy; to the vast network of interwoven freshwater aquifers, extensive lowlands, tropical and freshwater forests, and aquatic ecosystems; to its biodiversity with temperature, sunlight, and rainfall in an amount and combination that support cultivation and bountiful harvests of rice, sugar cane, yams, plantains, cassavas, oil palm, rubber, and timber. It is also a hunter and fisherman's delight as all the above are the natural occupations of the native people[1]. All these have given way because of the negative effect of oil exploration and gas flares.

The U.S. Department of Energy estimates that since 1960, there have been more than four thousand (>4000) oil spills, discharging several million barrels of crude oil into the ponds, ditches, creeks, beaches, streams, and rivers of the Niger Delta[1]. These have impacted negatively on the environment, economic life, and health of the people[2]. Gas flares, which have resulted in acid mists, have damaged plant and animals. Other problems rampant in the oil-rich region of Nigeria include illegal oil bunkering (crude oil theft), hostage taking of expatriate oil workers by splinter militant groups[3,4], and oil and gas pipeline vandalization. These have all affected Nigeria's image in the international community. Soil, water, and air contamination from oil pollution and gas flares has resulted in litigation between the host communities, oil companies, and governmental agencies, leading to claims and counter claims[5]. Over 2.5 billion $ft^3$ of crude-associated gas is flared in Nigeria daily with an estimated yearly financial loss of $2.5 billion. Skin lesions, cancer, accidents due to poor visibility, and malignant lymphomas have been reported in the Niger Delta region of Nigeria[6,7].

In this work, we intend to measure the acidity of rainwater from two major oil-producing states of the Niger Delta and compare that to levels of non-oil-producing and gas-flaring states. This is to ascertain if a correlation exists between gas flaring, pH values of precipitation, and numerous environmental and health problems in gas-flaring communities, so as to make a case for reinjecting gas rather than flaring by industry operators.

## MATERIALS AND METHODS

Rainwater samples were collected from Warri and Port Harcourt, two cities of the main oil- and gas-rich region of Nigeria's Niger Delta. (Port Harcourt is also noted for non-oil-manufacturing industries). Awka, a city outside the Niger Delta in the hinterland of southeastern Nigeria a few kilometers away from Onitsha and Nnewi, is known for several small- and medium-scale industries and is presented as the control. The rainwater was sampled between April–October 2005 and 2006, namely April-June 2005, July-August 2005, September-October 2005, April-June 2006, July-August 2006, and September-October 2006. In each city, the samples were collected from three points in a triangular equilibrium, using clean plastic basins fastened to a table, 2 m above ground level and 115 m away from tall buildings and trees. The water samples were filtered and acidity levels determined using a digital pH meter. The results of all the samples are an average of duplicate measurements for precision.

## RESULTS

The pH of rain samples from Port Harcourt and Warri were acidic when compared with those of Awka (control). The average pH values for the onset of rain, mid rain, and end of rain (April-June, July-August, and September-October) for Warri in 2005 and 2006 are as follows: 4.81, 4.70, 6.15, and 4.79, 4.80, 4.72 (Table 1) while that of Port Harcourt for the period and year of study are 4.71, 4.94, 4.93, and 5.04, 5.73, 4.91 (Table 2). The pH values from Awka (control) are 6.04, 5.88, 5.75, and 6.00, 5.10, 5.96 (shown in parentheses in Tables 1 and 2).

**TABLE 1**
**pH of Rainwater Samples in Warri between April and October, 2005 and 2006**

| Year | pH of Onset of Rain (April-June) | pH of Mid Rain (July-August) | pH of End of Rain (September-October) |
|---|---|---|---|
| 2005 | 4.81 ± 0.026 (6.04 ± 0.013) | 4.70 ± 0.017 (5.88 ± 0.009) | 6.15 ± 0.008 (5.75 ± 0.009) |
| 2006 | 4.79 ± 0.006 (6.00 ± 0.006) | 4.80 ± 0.056 (5.10 ± 0.048) | 4.72 ± 0.040 (5.96 ± 0.006) |

Data presented as mean ± SEM.

Values in parentheses represent control data from Awka city.

N = 8–15 corresponding to episodes of rainfall within the stipulated period.

**TABLE 2**
**pH of Rainwater Samples in Port Harcourt between April and October, 2005 and 2006**

| Year | pH of Onset of Rain (April-June) | pH of Mid Rain (July-August) | pH of End of Rain (September-October) |
|---|---|---|---|
| 2005 | 4.71 ± 0.003 (6.04 ± 0.013) | 4.94 ± 0.003 (5.88 ± 0.009) | 4.93 ± 0.005 (5.75 ± 0.009) |
| 2006 | 5.04 ± 0.006 (6.00 ± 0.006) | 5.73 ± 0.048 (5.10 ± 0.048) | 4.91 ± 0.006 (5.96 ± 0.006) |

## DISCUSSION

A consequence of oil exploration and exploitation in the Niger Delta region of Nigeria is the numerous gas flare points that accompany crude oil wellheads and refineries. The smoke that comes from the gas flares does not just contain sooty grey particles, but also many invisible gases that can be very harmful to our environment. These gases (especially nitrogen oxide and sulfur dioxide) react with the tiny droplets of water in clouds to form sulfuric and nitric acids. The rain from these clouds then falls as “acid rain”[8]. Although all rain tends to be acidic because of the equilibration of water with atmospheric carbon dioxide, yielding a pH of 5.6–6.5, precipitation with a pH below 5.0 is a result of a significant anthropogenic contribution of pollutants, mainly sulfur dioxide and nitrogen oxide[9].

In Nigeria, anthropogenic activities in the Niger Delta that may give rise to acid rain precursor gases are numerous. Sulfur dioxide ($SO_2$) can be produced by iron and steel production, smelting of metal sulfate ore, plankton, and rotting vegetation; are all common features of the study area. In many Nigerian rural areas as well as modern cities, worn out tires are used to roast poultry in the abattoirs, which release high amounts of $SO_2$ into the atmosphere. Oxides of nitrogen ($NO_x$) are by-products of firing processes of extremely high temperature (automobiles, utility plants) and in chemical industries (fertilizer production). Natural processes, such as bacterial action in soils, forest fires, and lightening, can contribute significantly to the high value of this acid-forming gas in the atmosphere.

Unfortunately, hitherto, the Nigerian government has not paid encouraging attention to environmental degradation. Nigeria is the highest importer of used vehicles, whose combustion efficiency is not guaranteed. According to the April 2006 edition of *African Review of Business and Technology,* London-based magazine survey, owing to power outages, Nigeria is the largest importer of diesel generators, estimated to cost $152 million/annum, with their attendant environmental and public health implications. Adverse health impacts from pollutants associated with goods movements include cancer risk, respiratory illnesses, and an increased risk of heart disease[10]. The survey focused on diesel-powered generators in the range of 1 to 200 kva and the figures keep rising. If gas-powered generators were included, the figures would triple, as many multinationals, institutions, homes, and small businesses depend on diesel and gas generators for their source of power[11]. The acid precursor gases ($SO_x$ and $NO_x$) that cause acid rain in the Niger Delta region may be transboundary pollutants[12]. Atmospheric reactions that lead to acid precipitation can take place in gas or aqueous phases. In the gas phase, photolysis is important in the daytime. The main oxidant for $SO_2$ conversion to sulfuric acid is hydroxyl radical ($OH^-$), represented as

$$SO_2 + OH \leftrightarrow HOSO_2$$

$$O_2 + HOSO_2 \leftrightarrow HO_2 + SO_3$$

$$SO_3 + H_2O \leftrightarrow H_2SO_4$$

Diurnal variation in OH makes the above reaction less important at night. Hydrogen peroxide is the principal oxidant in the aqueous phase, such as

$$SO_2 + H_2O \leftrightarrow H^+ + HSO^-_3$$

$$HSO^-_3 + H_2O_2 \leftrightarrow A^- + H_2O$$

$$A^- + H^+ \leftrightarrow H_2SO_4$$

where $A^-$ is intermediate specie[9]. Gas phase oxidation of nitrogen oxides to nitric acid is important in the daytime, with hydroxyl radical as principal oxidant and proceeds as

$$NO_2 \leftrightarrow NO+O^-$$

$$NO +O_3 \leftrightarrow NO_2 + O_2$$

$$NO + OH+m \leftrightarrow HNO_2$$

$$NO_2 +OH+m \leftrightarrow HNO_3$$

where m is any third body that can absorb energy, namely $N_2$ or $O_2$. At night, any ozone present would oxidize $NO_2$ to the nitrate radical, which in reacting with $N_2O$, would yield $N_2O_5$ and ultimately nitric acid[9]. By definition, the precipitation in the two major oil-producing cities of the Niger Delta region are acidic as measured between April and October of 2005 and 2006. With an average pH of 4.86, the precipitation of Port Harcourt was more acidic than that of Warri (5.22) in 2005. However, in 2006, the pH (4.77) of precipitation in Warri was more acidic than that of Port Harcourt (5.23). The acidity of rain in Warri in 2006 was noticeable in that it caught the attention of a foremost national daily in Nigeria[13]. Average pH levels for the 2 years under study (2005 and 2006) show that the rainfall in Warri was more acidic than that of Port Harcourt; for the 2 years (2005 and 2006), the pH of precipitation in Awka acting as control is within acceptable range. The acidity is such that it can cause serious damage to biota, despite the area being along the coast of the Atlantic Ocean and under the influence of moisture-laden southwesterly maritime air masses that bring rain to Nigeria and may have diluting effect on the pH[14]. Individual precipitation acidity (pH value) of the study area differs from the mean because of localized pollution.

The biodiversity of the Niger Delta is under serious threat; the worst affected are the interwoven water bodies. There have been reduced fish catches; plankton, freshwater shrimp, snails, and mussels on which fish feed are most affected by acidification. Fish such as minnows, salmon, and roach are equally threatened. Roe and fry (eggs and young) of the fish are the worst affected. The acidification can also cause deformity of young fish and can prevent eggs from hatching properly, which leads to fish-eating birds and animals also being affected[8]. Important cations, such as $K^+$, $Ca^{2+}$, $Mg^{2+}$, and $Na^+$, which are very important to the welfare of green plants and the aquatic ecosystem, are leached out and become unavailable to plants as nutrients, as well as demobilization of toxic cations into the aquatic ecosystem where they bioconcentrate in lugworms, barnacles, algae, and other planktonic and benthic organisms enroute to food webs[9].

Sulfates and nitrates, which form in the atmosphere from sulfur dioxide ($SO_2$) and nitrogen oxide ($NO_x$) emissions, contribute to visibility impairment. About 50–70% of visibility reductions in the eastern U.S. that negatively affect the economic values of national parks have been traced to sulfate particles, while nitrates and carbon play important roles in the western U.S.[15]. Acid precipitation can wear away the waxy protective coatings of leaves, damage them, and reduce photosynthesis. In the Niger Delta region, serious health problems such as skin cancers and lesions may be linked to acid rain. Stomach ulcers could also occur, as consumption of acidic water can alter the pH of the stomach and leach the mucous membrane of the intestinal walls. Nigerians depend heavily on rainwater for drinking, cooking, laundry, and other domestic uses. It is hoped that a potable water supply will become a high-priority issue for the government as water treatment is rarely carried out at the household level.

Statues (marble and bronze works), buildings, vehicles, pipes, cables, and textile materials are attacked by acidic solution (rain); galvanized steel and painted surfaces are corroded[9]. Noise and thermal pollution from numerous gas flare points and extensive fire disasters that characterize the study area have resulted in the extinction of some species that are hunters' delight. Inhalation of fine particulates that result from acid precursor gases have been linked to illness and premature death from heart and lung disorders, such as asthma and bronchitis[15]. Apart from acidic precipitation and its negative impact, acid rain precursor gases ($NO_2$ and $SO_2$) are part of six common outdoor pollutants[16]. Nitrogen dioxide ($NO_2$) poses a health threat itself as well as playing a major role in the formation of the photochemical pollutant ozone. Research has shown that animals exposed to $NO_2$ have diminished resistance to both bacterial and viral infection[17,18], while children exposed to high indoor levels of $NO_2$ may become more susceptible to critical infections of the lower respiratory tract, bronchial tubes, and lungs, and may develop bronchitis and chest cough with phlegm[19].

Sulfur dioxide ($SO_2$) is a temporary irritant, although studies have shown that increased levels of $SO_2$ in conjunction with particulate matter may trigger small, but measurable, temporary deficits in lung function[20,21]. Epidemiological studies have found that the impact of $SO_2$ is inseparable from that of particulate matter, but effects of the two classes of pollutant have been distinguished in a more recent analysis exemplified by studies in Utah Valley[22]. The major health impact of $SO_2$ is on population groups susceptible to pollutant effect due to pre-existing conditions, such as asthma. Clinical studies have demonstrated the rapid onset of bronchoconstriction in asthma patients exposed to relatively low (0.25–0.5 ppm) $SO_2$ levels[23,24,25]. Increased acidity of rain indicating high $NO_2$ and $SO_2$ emissions may predispose the natives to spontaneous abortion, ectopic pregnancy[26], malignant lymphomas[6,7], or soft tissues sarcomas[27].

The increased and extensive damage to the biota, litigations and counterlitigations, social crises and security risks are manifestations of a polluted and unhealthy environment. The government is aware of the effect of acid deposition on the health of the people and the environmental damage manifested in increasing damage to lakes, soils, plants, animals, forests, and fisheries[28], so laws were enacted to ensure atmospheric protection. Section 18 and 19 of the FEPA Act Cap. F10 laws of the Federation of Nigeria 2004 provide for air quality, atmospheric, and ozone protection and by Section 19 (2), the Agency may make recommendations and programs for the control of any substance, practice, process, or activity that may reasonably be anticipated to affect the atmosphere, especially ozone in the stratosphere, when such effect may reasonably be anticipated to endanger public health or welfare. Although this provision did not make pollution of the atmosphere a criminal offence, by the provision of Section 22 (1) (b) of the FEPA Act, it behooves the polluter of the atmosphere to bear the responsibility of restoring the environment in the form of cost to third parties either through reparation, restoration, restitution, or compensation, as maybe determined by the agency from time to time. The Associated Gas Re-injection (Amendment) Decree of 1985, by the provision of its Section 1, seems to be in tandem with the FEPA Act, by adopting the Polluter Pays principle or scheme. Unfortunately, this principle may not suffice because pollution such as air pollution could take a longer time to manifest and by the time it is discovered, its effect could have become devastating, as there may be no limit to the number of persons or extent of the environment affected, so the issue of paying compensation or restoring the atmosphere to its near natural state maybe "mission impossible". The best policy, therefore, would be to enforce the laws that already exist covering pollution abatement equipment installation and to ensure that the deadline given to gas flaring is maintained.

It must be noted that besides the common law remedies in tort of nuisance, negligence, and the rule in Ryland V Fletcher, not much work appears to have been done to expand the legal control of air and atmospheric pollution in Nigeria. Although Section 247 of the criminal code makes it a criminal offence to impair the atmosphere in such a way as to make it noxious to the health of persons in general dwellings or who conduct business in the neighborhood, and Section 1 of the Harmful Waste (Special Criminal Provision etc) Act makes it an offence to carry, deposit, dump, transport, import, sell or offer for sale, purchase, or deal in any harmful waste inimical to the atmosphere, there are a dearth of cases prosecuted on the basis of these provisions, thus making the existing laws perfunctory in nature as there is no enforcement mechanism to back up the statutory regulation in place[29]; more so standard of proof shall strictly be in accordance with the provisions of the Section 138 of the Evidence Act.

There are those who believe the attitude of the Nigerian court to be dismal and discouraging towards litigation relating to pollution in the oil industry[30]. This, however, may not be unconnected with the fact that the oil industry is the chief economic earner, since according to Duruigbo et al.[31], 80% of our revenue generation is realized from oil and oil-related activities accounting for over 95% of total exports and over 90% of the country's foreign exchange earnings. In Allar Irou V Shell BP Development Company Nigeria Limited, Unreported Suit No. W/89/71, Warri High Court (Judgment delivered on 26th No. 1973), the judge refused a successful plaintiff an order for injunction stating that such an order would be tantamount to ordering the defendant oil company to cease operations in the area and that, needless to say, mineral oil is the main source of this country's (Nigeria) revenue. Similarly, in Chinda V Shell BP (1974) 2 RSLE1, where the plaintiffs complained of adverse impact of "gas flare" on their buildings, farm crops, and other plants, and prayed that the court among others would restrain Shell BP from operating a flare stack within 5 mi of the plaintiffs' village, the judge refused to make an order of the injunction as asked and described the relief sought as "an absurd and needlessly wide demand". Condemning this kind of attitude to environmental claims, Ajomo rightly observed, as noted by Ukiri et al.[32], that what the judges failed to realize is that economic development can be compatible with environmental conservation. As the people of the area are wary of the government's tardiness and the insensitivity of oil company operators to issues relating to their environment, health, and social well-being, the Environmental Rights Action (ERA) in August 2005 Instituted a suit on behalf of eight communities (Rumuekpe, Imiringi, Gabrain, Eremah, Akala-olu, Idama, Iwherekan, and Eket) under the devastating effect of gas flare in one of the Niger Delta states against five oil companies (Shell Petroleum Development Company Nigeria Limited, Total/FINA/ELF Limited, Nigeria/Agip Oil Company Limited, Chevron/Texaco Nigeria Limited, and the Nigerian National Petroleum Corporations); also joined in the suit is the attorney-general of the Nigerian federation.

In the suit, the Counsel to the applicants sought the leave of the Court to apply for an order securing the enforcement of their fundamental rights to life and dignity of human persons as provided by Sections 33 (1) and 34 (1) of the constitution of the Federal Republic of Nigeria, 1999 and further secured by Articles 4, 16, and 24 of the African Charter on Human and Peoples Rights (Ratification and Enforcement)[5]. The relief sought by the applicant includes a declaration that:

> "the failure of the first to fifth defendants to carryout environmental impact assessment (EIA) in the applicants communities concerning the effects of their gas-flaring activities was a violation of section 2 (2) of the Environmental impact Assessment Act, Cap. E12, Vol. 6 laws of the Federation of Nigeria, 2004 and contributed to the violation of the applicant's fundamental rights to life and dignity of human person and to compel the companies to stop gas flaring in the entire Niger-Delta region"[5].

The applicants based their case on relevant statutes providing for compensation for oil pollution, such as the Oil Pipelines Act of 1955 Cap. 145, the Petroleum Act 1969, and Petroleum (Drilling, and Production) Regulation 1969[33]. Although the case is still pending at a Benin federal high court, its outcome may bring to an end the era of gas flaring. Gas flaring is a colossal waste if placed against gas reinjection, a reduction in greenhouse gas, acid rain, thermal pollution and the negative impact on the environment and public health, proper impact assessment, remediation of already polluted environment, and compensation to the affected communities.
